# Behavioral and neurophysiological aspects of working memory impairment in children with dyslexia

**DOI:** 10.1038/s41598-022-16729-8

**Published:** 2022-07-22

**Authors:** Jie Wang, Shuting Huo, Ka Chun Wu, Jianhong Mo, Wai Leung Wong, Urs Maurer

**Affiliations:** 1 Department of Psychology, The Education University of Hong Kong, Hong Kong S.A.R., China; 2 Department of Psychology, The Chinese University of Hong Kong, Hong Kong S.A.R., China; 3 Brain and Mind Institute, The Chinese University of Hong Kong, Hong Kong S.A.R., China; 4 Department of Educational Psychology, The Chinese University of Hong Kong, Hong Kong S.A.R., China

**Keywords:** Neuroscience, Psychology

## Abstract

The present study aimed to identify behavioral and neurophysiological correlates of dyslexia which could potentially predict reading difficulty. One hundred and three Chinese children with and without dyslexia (Grade 2 or 3, 7- to 11-year-old) completed both verbal and visual working memory (*n*-back) tasks with concurrent EEG recording. Data of 74 children with sufficient usable EEG data are reported here. Overall, the typically developing control group (*N* = 28) responded significantly faster and more accurately than the group with dyslexia (*N* = 46), in both types of tasks. Group differences were also found in EEG band power in the retention phase of the tasks. Moreover, forward stepwise logistic regression demonstrated that both behavioral and neurophysiological measures predicted reading difficulty uniquely. Dyslexia was associated with higher frontal midline theta activity and reduced upper-alpha power in the posterior region. This finding is discussed in relation to the neural efficiency hypothesis. Whether these behavioral and neurophysiological patterns can longitudinally predict later reading development among preliterate children requires further investigation.

## Introduction

Children with developmental dyslexia have normal intelligence and sufficient learning opportunities but experience unexpected difficulty in learning to read and write. Understanding the neurobiological basis of dyslexia might facilitate earlier and possibly more accurate identification of children who will develop dyslexia, which is critical for early and most effective intervention. So far the most commonly observed brain differences in people with dyslexia seem to be reduced activations in left temporal, parietal, and fusiform regions^1^. These studies mostly compared brain activations of people with and without dyslexia during a variety of reading or reading-related tasks, such as fluent reading^2^ and phonological awareness tasks^3^. However, few studies have investigated brain activations of people with dyslexia in a working memory (WM) task, although WM measures have been found to predict reading performance^4^.

WM is a system that temporarily stores and manipulates information we need in completing cognitive tasks^5^. In this model, visual and verbal information is maintained by domain-specific slave systems called the visuospatial sketch pad and the phonological loop, while the central executive acts as an attentional-controlling component and coordinates the manipulation of information from the slave systems. Backward digit or word span tasks are widely used to measure verbal WM, as this type of task requires participants to recall a sequence of digits or words in a reversed order from how they are presented (e.g., recalling “3–2–7” after hearing “7–2–3”). Here participants need to not only maintain the original sequence of verbal information but also manipulate it to generate a new sequence in the reversed order. Simultaneous storage and processing of information is a key characteristic of WM tasks, and essential for many cognitive skills. A review study has shown that there is a moderate correlation between WM and academic achievement including both reading and math performance^6^. Below we briefly review previous studies linking reading development and WM.

### Reading development and WM

WM is closely related to the concept of short-term memory, which refers to the capacity to store information for a short time interval. Verbal short-term memory is typically measured by forward span tasks that require participants to recall a sequence of digits or words in the same order as presented to them. Since there is no need to manipulate the information, short-term memory tasks are considered to tap into part of WM functions only. In a meta-analytic review^7^, children with dyslexia performed significantly worse than chronological age controls on verbal short-term memory tasks. However, when two other predictors (i.e., phonemic awareness and rime awareness) were controlled, verbal short-term memory did not contribute uniquely to children’s word reading performance.

Since WM measures reflect people’s ability to simultaneously maintain and process information, researchers have linked WM more frequently with reading comprehension, a more complex reading ability that involves integrating information from earlier text, current input, and one’s long-term memory. A review study has reported a moderate correlation between WM and reading comprehension^4^. Researchers have also investigated the association between WM and early reading development. For example, Chung and McBride-Chang^8^ found that WM and inhibitory control together contributed uniquely to word reading performance of Hong Kong Chinese children at both the second and third years of kindergarten, after controlling for age, vocabulary knowledge, and metalinguistic skills (see also the study of Welsh and colleagues^9^). These findings are consistent with a prevalent observation that individuals with dyslexia tend to show deficits in WM^10–12^.

Most reading studies have emphasized the role of verbal rather than visual working (or short-term) memory in predicting reading development. This is because the majority of reading studies have been conducted among readers of alphabetic scripts, in which grapheme-phoneme correspondences allow the readers to decode the sound of a spelling in a relatively easy manner^13^. However, the role of visual WM may be important in learning to read other types of scripts with a lack of grapheme-phoneme correspondences, e.g., Chinese. Since Chinese adopts a logographic writing system, children typically need to rely on rote character copying in Chinese literacy acquisition^14^. Hence, it is not surprising that visual skills seem to be more important for Chinese readers than for alphabetic readers. In a cross-cultural study, Huang and Hanley^15^ found that performance on a task of visual paired associates learning (i.e., learning the association between colors and abstract line drawings) was a strong predictor of the reading ability of 8-year-old primary children in Hong Kong and Taiwan (Chinese script), but not in Britain (English script). Later studies also found that visual skills contributed uniquely to Chinese character reading after controlling for age, vocabulary knowledge, and metalinguistic skills^16^.

Although researchers have compared Chinese children with and without dyslexia on a variety of visual skill measures^11^ (e.g., visual search, visual spatial relationship), few of them have adopted visual WM tasks. Therefore, the first aim of the present study was to fill in this research gap by using the *n*-back task^17^ with both visual and verbal stimuli. In this task, participants are typically presented a sequence of items one by one and required to indicate whether the current item is the same as the one presented *n* items back. For example, the second “3” in the sequence “5–2–3–**3**–0” is a target in a 1-back task, and the second “2” in the sequence “9–2–0–**2**–3” is a target in a 2-back task. As the sequence goes on, participants need to continuously update the information maintained in the WM and perform same-different judgment at the same time. WM demand varies with *n* and thus can be manipulated factorially. In the present study, two types of *n*-back task were used to compare Chinese children with and without dyslexia, one with Chinese characters as items while the other with visual patterns. These two types of tasks would reflect children’s verbal and visual WM, respectively. Another important reason for choosing the *n*-back task is that the procedure of this task fits the methodological constraints in many neuroimaging techniques, which is related to the second aim of the present study as spelled out below.

### Brain oscillations in relation to WM processes and individual differences

Despite the close relation between WM and reading development, few studies have investigated the potential neurophysiological substrate of a WM deficit in dyslexia. Nevertheless, the relation between brain oscillations and maintenance of information in WM has been extensively studied in the past few decades. A recent review^18^ has shown that theta activity in the frontal midline region is most consistently associated with WM maintenance, especially in verbal WM tasks. The most typical finding is an increase in frontal midline theta during a retention phase of the WM task relative to the baseline^19^. Among 15 EEG studies identified in the review that involved at least two load levels of verbal WM, ten reported stepwise increase of theta with memory load^20^. On the other hand, theta increase has been less consistently observed in visual WM tasks (around half of the reviewed studies).

Due to its commonly observed increase at higher task difficulties, frontal midline theta is considered to reflect the mental effort engaged in the task^21^. Hence, one may expect to see more pronounced theta increase in less apt individuals, since they probably need even more effort to complete a demanding task than more apt individuals do. This prediction is consistent with the neural efficiency hypothesis^22^, which posits that better performing individuals show reduced (more efficient) neural activity in cognitive tasks. In an EEG study by Maurer and colleagues^23^, healthy adults were required to memorize 2 or 4 unfamiliar symbols (low or high load) for 3.5 s and then to decide if a probe was one of the presented symbols (i.e., a Sternberg task^24^). The participants’ behavioral performance decreased from the low load condition to the high load condition, while their frontal midline theta in the retention phase increased. Moreover, the theta increase correlated significantly with the decrease in accuracy at the individual level, indicating that the more difficult a given task seemed to an individual (as indicated by the larger decrease in accuracy), the larger increase in frontal midline theta was observed. Another study by Brzezicka and colleagues^25^ recorded intracranial EEG while patients with epilepsy were performing a Sternberg task with 3 load levels (1, 2, or 3 pictures). Theta power in the retention phase increased with memory load in the hippocampus but decreased in the dorsolateral prefrontal cortex (DLPFC). Furthermore, the faster one participant responded, the larger theta power decrease with memory load was found in the DLPFC. Although the load effect on theta power was partly in different directions, both studies found that better performing participants showed a tendency of reduced theta power (smaller increase or larger decrease with memory load) in the retention phase of a WM task.

In addition to theta power, alpha power has also been found to change with WM load, although the direction of alpha change was inconsistent. According to the review by Pavlov and Kotchoubey^18^, the ratio of studies on verbal WM that found alpha increase versus decrease was 4:1, and this ratio became 3:2 for studies on visual WM. By dividing the alpha band into subbands (lower- and upper-alpha), some researchers found that upper-alpha activity tended to increase with memory load while lower-alpha activity tended to decrease^20,23^. One explanation is that upper-alpha increase reflects inhibition of task-irrelevant regions in the retention phase and that lower-alpha decrease reflects release of inhibition in task-relevant regions^20,26^. In terms of individual difference, Grabner and colleagues^27^ found that higher intelligence was associated with higher upper-alpha power (i.e., lower brain activation) in WM tasks, consistent with the neural efficiency hypothesis^22^.

### The present study

So far, much fewer studies have investigated WM-related brain oscillations in children^28^, and almost none for children with dyslexia^29^. The present study recorded concurrent EEG while the children with and without dyslexia were doing the *n*-back task, in order to compare not only behavioral performance but also brain oscillations of the two groups in a WM task. In addition to verbal WM, nonverbal stimuli were also included to tap visual WM (considering the logographic nature of the Chinese writing system), which seemed to have received less attention in the reading literature. Based on the previous findings mentioned above, we chose to examine theta power in the frontal midline region^19,21^ as well as lower- and upper-alpha power^23^ in the posterior region^30,31^. Moreover, logistic regression analysis was conducted to find out whether these behavioral and neurophysiological measures were significant predictors of dyslexia^32,33^. We hypothesized that the children with dyslexia would show poorer behavioral performance in the WM task. The neural efficiency hypothesis predicted that the children with dyslexia would manifest higher theta and lower alpha power (i.e., more brain activation) during the retention phase of the task.

## Results

In the current design, Type of task (verbal, visual) and Load level (1-back, 2-back) were two within-participants factors, while Group (control, dyslexic) was a between-participants factor. Since the control group had a significantly higher non-verbal intelligence than the group with dyslexia (*p* = 0.014), ANCOVA was conducted on each of the dependent variables in the *n*-back task with Intelligence as a covariate: (1) reaction time (RT, i.e., the time interval between stimulus offset and response) when the target was correctly hit, (2) *d* prime^17^, which was calculated based on the hit and false alarm rates, (3) log-transformed theta power in the frontal midline region, (4) log-transformed lower-alpha power in the posterior region, and (5) log-transformed upper-alpha power in the posterior region. The restriction to the frontal midline region for theta analysis was based on previous studies that showed a topographically more restricted effect for theta compared to alpha^20,23^, while alpha activity was relatively widespread and most prominent over posterior areas^30,34^.

### *d* prime

Calculated based on the hit and false alarm rates, *d* prime (*d'*) can be considered as an index reflecting how well one can differentiate targets from non-targets (the higher *d'*, the better). Table 1 shows the mean *d'* in each of the four conditions for each group. The ANCOVA showed that the main effect of Intelligence on *d'* was significant (*F*_(1,71)_ = 6.30, *p* = 0.014, *ƞ*_*p*_^2^ = 0.08) while all interactions involving this covariate were non-significant (*p*s ≥ 0.150). The main effects of Type (*F*_(1,71)_ = 14.87, *p* < 0.001, *ƞ*_*p*_^2^ = 0.17), Load (*F*_(1,71)_ = 5.23, *p* = 0.025, *ƞ*_*p*_^2^ = 0.07), and Group (*F*_(1,71)_ = 15.80, *p* < 0.001, *ƞ*_*p*_^2^ = 0.18) were significant, showing that the *d'* was higher in the verbal tasks than in the visual tasks and also higher in the 1-back tasks than in the 2-back tasks. Besides, the control group demonstrated a higher sensitivity to detect the targets in the WM tasks than the group with dyslexia. The Load × Group interaction (*F*_(1,71)_ = 4.90, *p* = 0.030, *ƞ*_*p*_^2^ = 0.07) and the Type × Load × Group interaction (*F*_(1,71)_ = 4.84, *p* = 0.031, *ƞ*_*p*_^2^ = 0.06) were significant, while all other interactions were non-significant (*p*s ≥ 0.369). The Load effect was comparable between the two groups in the verbal tasks (− 0.54 vs. − 0.58, *p* = 0.863, *d* = 0.04). In the visual tasks, the control group showed a significantly larger Load effect than the group with dyslexia (− 1.53 vs. − 0.77, *p* = 0.001, *d* = − 0.80), possibly because the group with dyslexia performed poorly in the visual 1-back task already.

**Table 1:** Behavioral performance and EEG band power of the typically developing and dyslexic children in the n-back task (standard errors in parentheses).

	Verbal 1-back		Verbal 2-back		Visual 1-back		Visual 2-back	
	*M*	*SE*	*M*	*SE*	*M*	*SE*	*M*	*SE*
***d* prime**								
Control	3.49	(0.16)	2.95	(0.20)	2.84	(0.22)	1.31	(0.12)
Dyslexic	2.66	(0.16)	2.08	(0.14)	1.71	(0.14)	0.94	(0.11)
All	2.98	(0.12)	2.41	(0.12)	2.14	(0.14)	1.08	(0.08)
**Reaction time (ms)**								
Control	779	(33)	940	(46)	830	(36)	1044	(56)
Dyslexic	932	(36)	1173	(36)	1095	(45)	1172	(61)
All	874	(27)	1085	(31)	995	(34)	1123	(44)
**Frontal midline theta power (log-transformed)**								
Control	0.546	(0.037)	0.531	(0.041)	0.583	(0.044)	0.553	(0.036)
Dyslexic	0.566	(0.028)	0.588	(0.030)	0.583	(0.029)	0.588	(0.030)
All	0.559	(0.022)	0.566	(0.024)	0.583	(0.024)	0.575	(0.023)
**Upper-alpha power in the posterior region (log-transformed)**								
Control	0.744	(0.057)	0.731	(0.055)	0.743	(0.060)	0.713	(0.059)
Dyslexic	0.579	(0.056)	0.573	(0.054)	0.590	(0.053)	0.595	(0.053)
All	0.642	(0.042)	0.633	(0.040)	0.648	(0.041)	0.639	(0.040)
**Lower-alpha power in the posterior region (log-transformed)**								
Control	0.607	(0.049)	0.596	(0.044)	0.585	(0.046)	0.568	(0.049)
Dyslexic	0.547	(0.046)	0.546	(0.042)	0.548	(0.042)	0.557	(0.043)
All	0.570	(0.034)	0.565	(0.031)	0.562	(0.031)	0.561	(0.032)

### Reaction time

The ANCOVA showed that the main effect of Intelligence on RT and interactions involving this covariate were all non-significant (*p*s ≥ 0.434). The main effect of Group (*F*_(1,71)_ = 12.82, *p* = 0.001, *ƞ*_*p*_^2^ = 0.15) and the Type × Load × Group interaction (*F*_(1,71)_ = 4.56, *p* = 0.036, *ƞ*_*p*_^2^ = 0.06) were significant, while all the other effects were non-significant (*p*s ≥ 0.395). The group with dyslexia showed a slightly larger Load effect than the control group in the verbal tasks (241 vs. 162 ms, *p* = 0.129, *d* = 0.37). In the visual tasks, the Load effect of the group with dyslexia was marginally smaller (77 vs. 215 ms, *p* = 0.079, *d* = − 0.43), possibly because this group responded quite slowly in the visual 1-back task already. Post-hoc *t*-tests showed shorter RT in the control group than in the group with dyslexia for all the conditions (verbal 1-back: *p* = 0.005, *d* = − 0.69; verbal 2-back: *p* < 0.001, *d* = − 0.96; visual 1-back: *p* < 0.001, *d* = − 0.99) except the visual 2-back one (*p* = 0.129, *d* = − 0.34).

### Frontal midline theta

**Figure 1:**
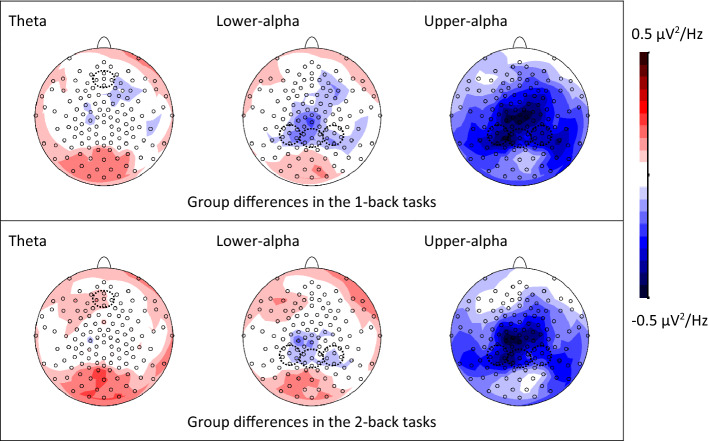
The group effects (dyslexic–control) on the log-transformed power of theta, lower-alpha, and upper-alpha bands during working memory maintenance. Data were collapsed across the two types of working memory tasks (verbal and visual). Frontal midline theta power was pooled from AFz plus 4 surrounding electrodes; posterior alpha power was pooled from Pz, P3, P4 and 15 surrounding electrodes (selected electrodes are enclosed by the dashed circles).

In the *n*-back task, the children needed to make same-different judgment for each presented item and to maintain the newly updated items before seeing the next one. The mean RT in each condition ranged from 779 to 1173 ms. We can infer that the last 1-s interval of the 3.5-s fixation period fell into the retention phase of the *n*-back task, before which the encoding of new information and the same-different judgment were completed. Thus, band power during WM maintenance was obtained from this time window (see Fig. 1 and Fig. S1 for the topographic maps of band power differences between groups and between load levels, respectively).

The ANCOVA showed that the main effect of Intelligence on log-transformed theta power and interactions involving this covariate were all non-significant (*p*s ≥ 0.223). The Load × Group interaction (*F*_(1,71)_ = 5.99, *p* = 0.017, *ƞ*_*p*_^2^ = 0.08) was significant, while all the other effects were non-significant (*p*s ≥ 0.318). Post-hoc *t*-tests showed that the log-transformed theta power of the control group had a marginally significant decrease from 1-back tasks to 2-back tasks (0.542 − 0.564 = − 0.022, *p* = 0.078, *d* = − 0.35). In contrast, the group with dyslexia demonstrated an opposite trend (0.588 − 0.575 = 0.013, *p* = 0.173, *d* = 0.20). In terms of group difference, the group with dyslexia had a non-significantly higher theta power than the control group in both 1-back and 2-back tasks (1-back: 0.575 − 0.564 = 0.011, *p* = 0.818, *d* = 0.06; 2-back: 0.588 − 0.542 = 0.046, *p* = 0.325, *d* = 0.24).

### Upper-alpha in the posterior region

The ANCOVA showed that the main effect of Intelligence on log-transformed upper-alpha power and interactions involving this covariate were all non-significant (*p*s ≥ 0.100). The main effect of Group (*F*_(1,71)_ = 4.91, *p* = 0.030, *ƞ*_*p*_^2^ = 0.07) was significant, while all the other effects were non-significant (*p*s ≥ 0.188). The control group demonstrated a higher upper-alpha power in the posterior region than the group with dyslexia.

### Lower-alpha in the posterior region

The ANCOVA showed that all the effects were non-significant on log-transformed lower-alpha power (*p*s ≥ 0.119).

### Distinguishing children with and without dyslexia

To find out which variables were most strongly and uniquely associated with dyslexia, the following variables from the WM tasks were entered by block^32^ into logistic regression models as predictors of dyslexia: (1) Block 1 included general control variables: age, grade, non-verbal intelligence; (2) Block 2 included 8 behavioral variables: reaction time and *d* prime in each of the four conditions; and (3) Block 3 included 12 neurophysiological variables: log-transformed frontal midline theta, posterior lower- and upper-alpha power in each of the four conditions. The forward Wald method^33^ was adopted in each block so as to identify behavioral measures (if any) that uniquely predicted dyslexia beyond control variables as well as unique neurophysiological predictors (if any) beyond both control and behavioral variables.

Table 2 shows the three logistic regression models generated in each block. In Block 1, the Wald statistic showed that only non-verbal intelligence was a significant predictor (*p* = 0.040). Model 1 made significantly better prediction of dyslexia than a null model (*χ*^2^_(1)_ = 4.99, *p* = 0.025). In Block 2, two behavioral variables (i.e., RT in the visual 1-back condition, *d'* in the verbal 2-back condition) were entered (*p*s ≤ 0.009), and Intelligence became non-significant (*p* = 0.328). Model 2 significantly improved prediction of dyslexia relative to Model 1 (*χ*^2^_(2)_ = 24.53, *p* < 0.001). The classification accuracy increased from 59.5% (dyslexic: 82.6%; control: 21.4%) to 81.1% (dyslexic: 84.8%; control: 75.0%), and Nagelkerke’s *R*^2^ improved from 0.089 to 0.448. In Block 3, two neurophysiological variables (i.e., log-transformed frontal midline theta in the verbal 2-back condition, log-transformed posterior upper-alpha in the visual 1-back condition) were further entered (*p*s ≤ 0.006), while the two behavioral variables remained significant (*p*s ≤ 0.002). Model 3 significantly improved prediction of dyslexia relative to Model 2 (*χ*^2^_(2)_ = 16.27, *p* < 0.001). The classification accuracy further increased from 81.1 to 82.4% (dyslexic: 87.0%; control: 75.0%). Nagelkerke’s *R*^2^ of Model 3 was 0.628, suggesting a relatively strong relation between the predictors and dyslexia (see Fig. S2 for the distribution of predicted probabilities in the classification plots). To sum up, significant and unique predictors of dyslexia were found from both behavioral and neurophysiological measures of WM in the present study.

**Table 2:** Parameter estimates, standard errors, and statistical significance in the logistic regression analyses of factors associated with dyslexia.

	*B*	*SE*	Wald	*p*	*χ*^2^	*df*	Nagelkerke’s *R*^2^	Classification accuracy
**Model 1**					4.99*	1	0.089	59.5%
Non-verbal intelligence	− 0.109	0.053	4.207	0.040*				
**Model 2**					29.52***	3	0.448	81.1%
Non-verbal intelligence	− 0.069	0.071	0.956	0.328	(Model 2 vs: Model 1: *χ*^2^_(2)_ = 24.53, *p* < 0.001)			
RT (visual 1-back)	0.005	0.002	10.548	0.001**				
*d'* (verbal 2-back)	− 0.889	0.339	6.872	0.009**				
**Model 3**					45.79***	5	0.628	82.4%
Non-verbal intelligence	− 0.048	0.094	0.260	0.610	(Model 3 vs: Model 2: *χ*^2^_(2)_ = 16.27, *p* < 0.001)			
RT (visual 1-back)	0.006	0.002	11.044	0.001**				
*d'* (verbal 2-back)	− 1.458	0.477	9.355	0.002**				
Theta (verbal 2-back)	7.098	2.582	7.559	0.006**				
Upper-alpha (visual 1-back)	− 4.998	1.735	8.302	0.004**				

*p < 0.05, **p < 0.01, ***p < 0.001.

**Figure 2:**
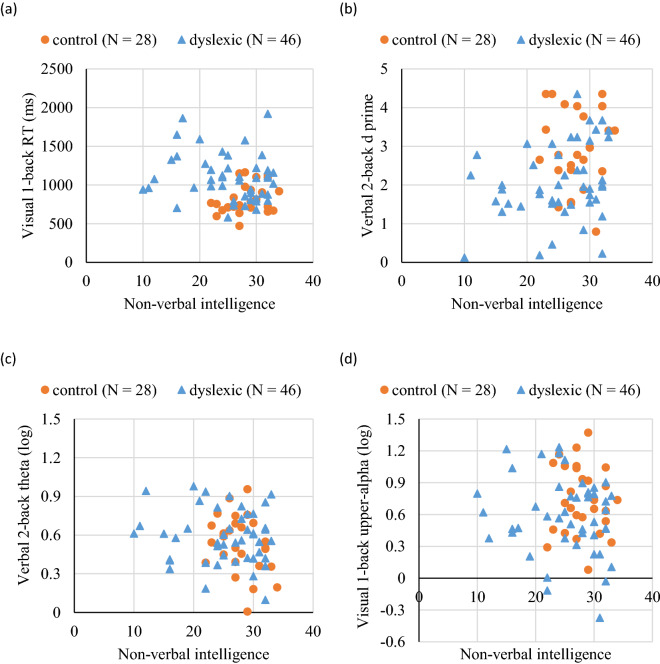
Scatterplots of the two groups showing each significant predictor of dyslexia (y axis) as a function of non‐verbal intelligence (x axis). Significant predictors included (**a**) reaction time in the visual 1-back condition, (**b**) *d'* in the verbal 2-back condition, (**c**) log-transformed frontal midline theta in the verbal 2-back condition, and (**d**) log-transformed posterior upper-alpha in the visual 1-back condition.

Figure 2 displays the scatterplots of the two groups, showing each significant predictor of dyslexia (y axis) as a function of non‐verbal intelligence (x axis). To further examine the predictive role of WM measures in dyslexia without any confounding effect of non‐verbal intelligence, we conducted additional logistic regression analysis on the control group and a subsample of the group with dyslexia (i.e., excluding those who scored 21 or lower in non‐verbal intelligence), whose non‐verbal intelligence scores were comparable (*p* = 0.860). The results showed that non‐verbal intelligence was no longer a significant predictor while behavioral and neurophysiological measures of WM (i.e., verbal 2-back RT, visual 1-back *d'*, verbal 2-back theta and upper-alpha) still significantly and uniquely predicted dyslexia. More details of the additional logistic regression analysis can be found in the Supplemental Material.

## Discussion

The present study required Chinese children with and without dyslexia to complete verbal and visual WM tasks with concurrent EEG recording. Consistent with the findings of previous studies^10–12^, the control group responded significantly faster and more accurately than the group with dyslexia, in both types of tasks. In the retention phase of the tasks, the frontal midline theta power of the control group tended to decrease from the low load to the high load condition, while the group with dyslexia showed an opposite trend. The control group also demonstrated a higher upper-alpha power than the group with dyslexia did across all conditions. Forward stepwise logistic regression identified a few significant predictors of dyslexia, including both behavioral and neurophysiological measures of WM.

### WM-related neurophysiological correlates of dyslexia

In the logistic regression model, frontal midline theta activity in the verbal 2-back task was positively associated with dyslexia, and posterior upper-alpha power in the visual 1-back task was negatively associated with dyslexia. This means that children in our sample who spent more mental effort and inhibited brain activation to a lesser extent during WM maintenance tended to have dyslexia, which is consistent with the neural efficiency hypothesis^22^. Importantly, these neurophysiological correlates uniquely predicted reading difficulty in addition to non-verbal intelligence (though non-significant in the final model) and behavioral correlates. While behavioral indicators (speed and accuracy) reflected the children’s final achievement in WM tasks, neurophysiological measures (theta and alpha oscillations) reflected the amount of mental effort and neural resources engaged during WM maintenance^21,23^. These variables tapped into different aspects of WM, and all predicted reading difficulty uniquely.

A few fMRI studies have also compared brain activation of dyslexic and non-dyslexic readers during WM tasks. While some found reduced prefrontal activation in dyslexia^35^, some found both increased and decreased activation in the prefrontal cortex^36^. These mixed results could be partly due to the differences in the tasks. Researchers have found that neural efficiency could be modulated by task difficulty and other factors^37^. There are situations where better performing individuals may display the same amount of or even more brain activation^38^. Besides, the relatively low temporal resolution of the fMRI technique could be another reason. An EEG study by Jaušovec and Jaušovec^39^ showed that in a modified *n*-back task, the theta power of high-intelligence individuals was higher than that of low-intelligence ones in 0–500 ms post-stimulus onset. Then it decreased soon and the group difference was reversed in 1000–2000 ms, suggesting that the high-intelligence individuals were more intensely engaged with the task in the first 500 ms and soon lowered their mental effort in the following retention phase. Hence, the retention phase, the phase selected for analysis in the present study, might be an optimal time interval for reduced neural activation in better performing individuals (i.e., neural efficiency) to occur. Previous fMRI studies might have captured neural activation across different phases, which could be one of the reasons for the mixed findings. By using EEG, the present study was able to examine brain activation in the retention phase more precisely.

In contrast to the prominent Group effect, the Load effect on brain oscillations seemed to be relatively minor in the present study. For frontal midline theta power, only the Load × Group interaction was significant, caused by the opposite directions of theta change in the two groups with increasing load. While the control group had a marginally significant theta decrease, the group with dyslexia demonstrated a non-significant trend of theta increase. For lower- and upper-alpha power, no significant difference was found between load levels. Different patterns of theta and alpha changes with WM load have been reported in previous studies, and the modulating factors remain largely unclear^18^. For example, a few studies found an inverted U-shape influence of WM load on frontal midline theta power, i.e., highest theta power with a moderate load level^40,41^. So, one explanation for the trend of theta decrease in the control group could be that the current 2-back tasks were beyond the moderate load level for early grade children. However, this account would predict a theta decrease in the group with dyslexia as well, since their behavioral performance was even worse than that of the control group. This prediction contradicted the observed trend of theta increase in the group with dyslexia. Note that the Load effect was not significant in either group. Further studies are needed to examine the robustness of the observed theta change when children perform WM tasks with different load levels.

### WM impairment: cause or effect

The present study identified a few behavioral and neurophysiological correlates (i.e., visual 1-back RT and upper-alpha, verbal 2-back *d'* and theta) of WM impairment in dyslexia (i.e., more neural resources engaged but poorer behavioral performance). However, we cannot tell whether poor WM is a cause or an effect of dyslexia. In the verbal *n*-back tasks, the children were able to make use of phonological codes of the Chinese characters to achieve better performance than in the visual tasks, where only visual codes were usable. The group differences in the verbal tasks could result from inefficient processing of Chinese characters in the children with dyslexia, due to their poor orthography–phonology conversion^42^. Hence, poor verbal WM could be a consequence of other deficits in dyslexia.

On the other hand, similar group differences were observed in the visual *n*-back tasks, where non-verbal stimuli were used without involving orthographic or phonological encoding. The visual 1-back RT and upper-alpha were also unique predictors of dyslexia in the logistic regression model. This finding seems to support a deficit in the central executive component of WM^43,44^, whose dysfunction impairs both verbal and visual working memories. Nevertheless, an alternative explanation is that the development of visual processing is influenced by the children’s reading skills^45^. In a longitudinal study, Pan and colleagues^42^ tracked Chinese children’s character reading accuracy and pure visual skill for three years (at ages 6 to 8). They found that reading accuracy predicted subsequent performance in a pure visual task but not vice versa. Hence, poor visual WM could be a consequence of reading difficulty as well.

Despite these alternative possibilities, we believe that the children with dyslexia are highly likely to have central executive dysfunction given the available evidence. Melby-Lervåg and colleagues^7^ have shown that verbal short-term memory does not contribute uniquely to word reading performance when metalinguistic skills are controlled. But WM together with other executive functions seems to have a unique contribution to reading achievement^8,9,46^, suggesting the importance of central executive in reading development. In the present study, similar group differences were observed across the two types of WM tasks, consistent with the existence of central executive dysfunction in dyslexia. Note that developmental dyslexia can be classified into different subtypes^47,48^, so not all children with dyslexia have the same deficits or the same cause of the deficits. The scatterplots in Fig. 2 show that not all children in the dyslexic group appeared to have WM impairment and that some control children demonstrated poor WM. Although WM differences can be observed in between-groups comparisons, WM impairment does not always co-occur with dyslexia^49^. In the current logistic regression analysis, Model 3 only correctly classified 87.0% of the children with dyslexia and 75.0% of the control children. Hence, behavioral and neurophysiological measures of WM could potentially predict dyslexia, but these measures alone are insufficient to classify children as having dyslexia or not.

The present study adopted the *n*-back task and identified both behavioral and neurophysiological correlates of WM impairment in dyslexia, among early grade children. Importantly, EEG band power (theta and alpha oscillations) uniquely predicted reading difficulty in addition to the behavioral measures (RT and *d'*). One limitation of the present study is that the non-verbal intelligence of the two groups was not matched, although additional logistic regression analysis on the subsample showed a similar pattern of results after matching the non‐verbal intelligence scores. Besides, previous studies found that dyslexic children’s deficits in certain short-term memory tasks disappeared when they were matched to controls on non-verbal intelligence and oral language abilities^50,51^. Although the current *n*-back tasks required key-pressing responses only and involved the use of oral language minimally (especially the visual tasks), this study could be improved by including measures of oral language abilities. With stricter control on non-verbal intelligence and oral language abilities, future studies may replicate the present study among younger or preliterate children and follow them up^52^ to find out whether earlier behavioral and neurophysiological measures of WM longitudinally predict later reading development. Compared to the backward span task and some other WM tasks, the *n*-back task has at least two advantages. First, the children are able to respond by simply pressing a key without verbal articulation, so that any confounding effects of language abilities are minimized. Second, the procedure of this task allows concurrent EEG recording and frequency analysis in a retention phase, which could potentially improve the prediction success. Longitudinal studies are needed to examine the effectiveness of these potential predictors and to determine whether they can be used in clinical practice.

## Conclusions

In the 1-back and 2-back WM tasks, the typically developing children performed better than the children with dyslexia. Frontal midline theta and posterior upper-alpha power in the retention phase of the tasks reflected the amount of mental effort and neural resources being engaged, and they predicted dyslexia uniquely in addition to indices of speed and accuracy. However, it remains unclear whether these behavioral and neurophysiological patterns are merely consequences of reading difficulty or not. Further investigation is needed to examine whether the current measures can be used to predict reading difficulty in pre-readers.

## Methods

### Participants

Data of 28 typically reading children and 46 children with dyslexia (native Cantonese-speaking; second or third grade; 7–11 years old) from a larger research project (approved by The Joint Chinese University of Hong Kong—New Territories East Cluster Clinical Research Ethics Committee) are reported. They were recruited from Hong Kong primary schools and education authorities, and written informed consent was obtained from the children and their guardians. All methods were performed in accordance with relevant guidelines. Among 103 children (66 with dyslexia) who completed all the relevant tasks as described below, 29 children (20 with dyslexia) were excluded from the present study due to too few usable EEG segments (see section on EEG recording and preprocessing below). The typically developing children had no difficulty in reading or writing based on parents’ report. Those with dyslexia were formally diagnosed by either educational or clinical psychologists based on The Hong Kong Test of Specific Learning Difficulties in Reading and Writing for Primary School Students—Third Edition [HKT-P(III)]^53^, whose criteria included adequate IQ (higher than 85), poor literacy (− 1 *SD* or below), and at least one area of cognitive-linguistic deficit (− 1 *SD* or below)^54^. Besides, they had no history of significant sensory impairment, birth complications, or brain injury. Based on the parents’ responses in a questionnaire (*N* = 70), 7 children from the dyslexic group (16.7%) and 2 from the control group (7.1%) had a formal diagnosis of language impairment (*p* = 0.244). Table 3 shows the demographic information of the two groups, who did not differ significantly in gender, age, grade, maternal or paternal education level, or monthly family income (*p*s ≥ 0.248).

**Table 3:** Demographic and other information.

Characteristic	Control	Dyslexic	*p*
Male-to-female ratio	16:12	22:24	0.437
**Age in months**			
*M*	103.6	101.3	0.248
*SE*	1.4	1.3	
*N*	28	46	
**Grade**			
*M*	2.71	2.72	0.977
*SE*	0.09	0.07	
*N*	28	46	
**Maternal education**			
*M*	2.82	3.07	0.407
*SE*	0.22	0.19	
*N*	28	44	
**Paternal education**			
*M*	3.00	2.93	0.829
*SE*	0.24	0.21	
*N*	27	43	
**Family income**			
*M*	4.04	3.93	0.777
*SE*	0.28	0.24	
*N*	28	43	
**Non-verbal intelligence**			
*M*	27.96	25.15	0.014*
*SE*	0.62	0.93	
*N*	28	46	
**Individual alpha frequency**			
*M*	9.36	9.22	0.438
*SE*	0.14	0.11	
*N*	28	46	

Coding of educational levels: 1 = middle school or below, 2 = high school, 3 = preparatory, 4 = college, 5 = postgraduate; monthly family income: 1 = HKD10,000 (USD1280) or below, 2 = HKD10,001–20,000 (USD1281–2560), 3 = HKD20,001–30,000 (USD2561–3840), 4 = HKD30,001–40,000 (USD3841–5120), 5 = HKD40,001–50,000 (USD5121–6400), 6 = HKD50,001 (USD6401) or above.

*p < 0.05.

### Procedure

#### Raven’s Standard Progressive Matrix (RSPM)

RSPM^55^, Sets A to C were used to assess non-verbal intelligence. Each test item required the children to choose an option out of six (Sets A and B) or eight (Set C) to fill the missing part of a design. There were 12 test items in each set and thus 36 items in total.

#### Verbal n-back task

**Figure 3:**
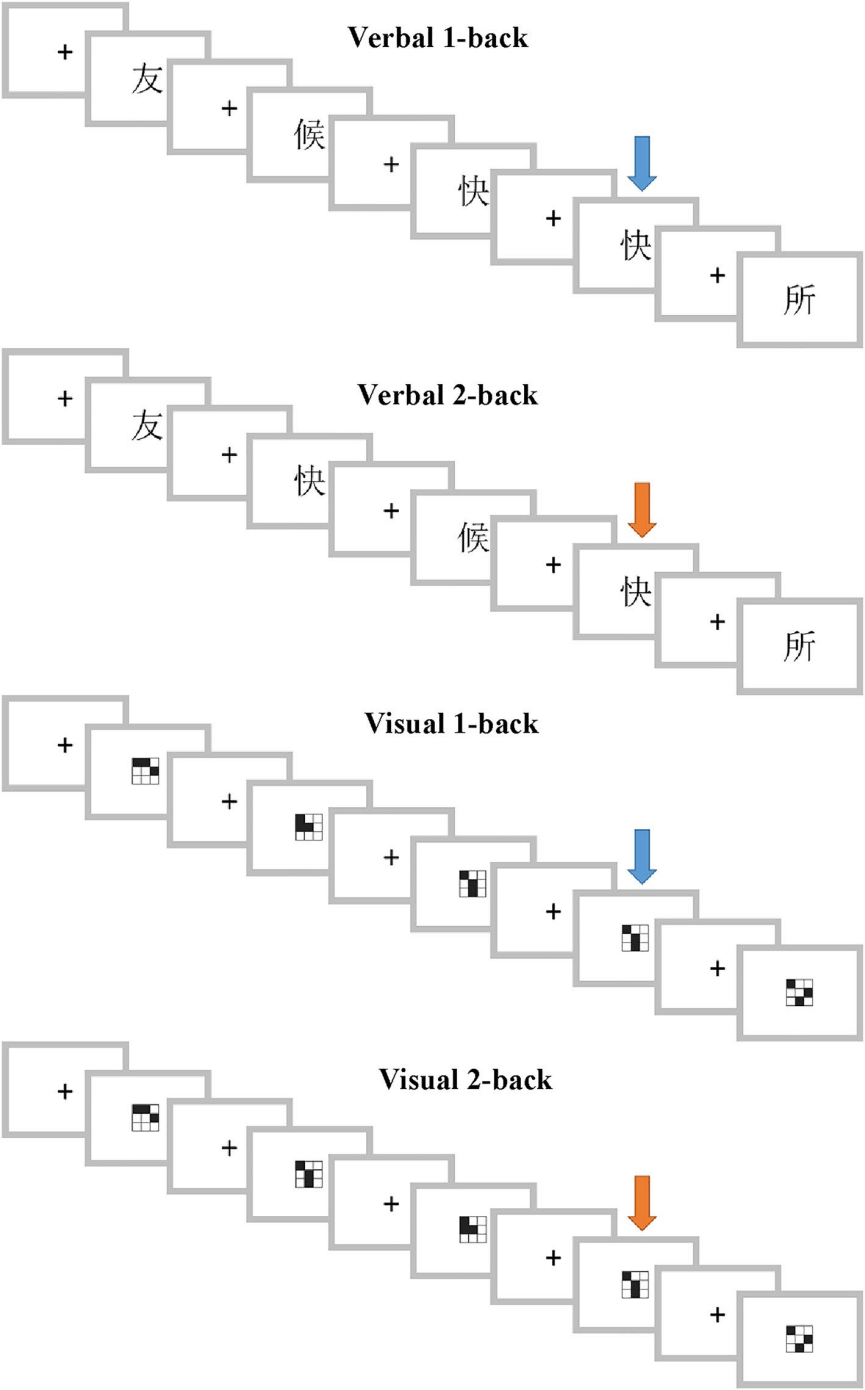
Example stimuli in the *n*-back task. Targets in each task are marked by arrows here for illustration.

Sixty Grade-2 level characters with a varying number of strokes (from 4 to 13) were selected from the Hong Kong Corpus of Primary School Chinese^56^ and formed a sequence for verbal 1-back and 2-back tasks respectively. In each task, twenty of the characters appeared twice, and those appearing at the second time were targets. The targets appeared immediately after the same characters in the 1-back task and appeared the second after the same characters in the 2-back task (Fig. 3). The same set of characters were used in both tasks, but the target characters were mostly different so that one could not predict whether a character was a target or not based on its status in an earlier task. Each task contained 60 non-targets and 20 targets and was divided into two blocks of 40 characters. The characters were sequenced in a way that there was no obvious semantic relatedness or orthographic similarity between each pair of consecutive characters, except for the targets in the 1-back task.

The *n*-back task was administered with the E-Prime 3.0 software (https://pstnet.com/products/e-prime/). Each character appeared for 500 ms, followed by a 3500-ms fixation and then the next character. The children needed to press “1” on the keyboard as accurately and quickly as possible, when they detected a target, and did not need to press any key for non-targets. At the beginning of each task, the task requirement (1-back or 2-back) was explained to the children, and they needed to complete a practice block with 14 characters (4 targets) to make sure that they understood the task requirement. They were able to repeat the practice once or more if needed.

#### Visual n-back task

Thirty 3 × 3 checkerboard patterns were created for visual 1-back and 2-back tasks. Each pattern contained 3 black squares and 6 white ones (see Supplemental Material). We did not use 60 different patterns, as it would yield too many similar patterns making the task too difficult. Similar to the verbal *n*-back task, both visual 1-back and 2-back tasks consisted of two blocks of 40 items (30 non-targets and 10 targets each). The patterns were sequenced in a way that each pair of consecutive patterns did not look similar, except for the targets in the 1-back task. The procedure was the same as that of the verbal *n*-back task. Each child completed all the four tasks (2 types × 2 load levels), whose order was counterbalanced across participants for each group.

#### EEG recording and preprocessing

**Figure 4:**
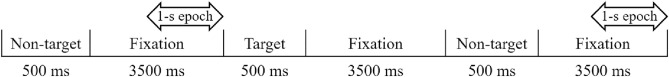
Parameters in the *n*-back task. EEG epochs in the last 1-s interval of the fixation period following non-targets were used in the frequency analysis.

EEG data were recorded at 500 Hz (online filter: 0.1–100 Hz; Cz as recording reference) using an EGI (Electrical Geodesics, Inc.) 128-channel system while each child was completing the *n*-back task in a quiet room. Impedances were controlled below 50 kΩ. The EEG recordings were offline filtered (0.1–70 Hz; notch filter: 50 Hz). Bad channels were excluded, and the remaining data were submitted to independent component analysis for eye movement correction^57^. The excluded channels were then spline interpolated^58^. All data were re-referenced to the average reference^59^ and then segmented to include the last 1-s interval of the fixation period (i.e., before the onset of the next item; see Fig. 4). Only segments following non-targets (without motor artefacts from key pressing) and not exceeding ± 100 μV were used in further analysis. Twenty-nine children with poor EEG data quality in any of the four conditions were excluded from the present study, so that all the 74 children reported here had no fewer than 10 usable epochs in each condition. The average number of usable EEG epochs was 29, 28, 31, and 29 in the verbal 1-back, verbal 2-back, visual 1-back, and visual 2-back conditions respectively.

Before the *n*-back task, EEG recordings were also obtained during a 3-min eyes-closed resting state block, with the same setting. The resting EEG data went through similar preprocessing steps and were segmented into 1-s epochs for determination of individual alpha frequency^60^ (IAF). The fast Fourier transformation was applied to each of the EEG epochs obtained from the *n*-back task and the eyes-closed state. For each child, the frequency with peak power density in the alpha band (8–13 Hz) across all electrodes in the eyes-closed state was identified as IAF. For two children, the alpha peak failed to be detected with power density averaged across electrodes, and power density at the occipital electrode Oz was then used to identify IAF; for another two children with eyes-closed EEG data of poor quality, their IAF was set at 10. The two groups did not differ significantly in IAF (*p* = 0.438).

Three frequency bands were defined with IAF as an individual anchor point: (IAF-6) to (IAF-3) as theta band, (IAF-2) to (IAF-1) as lower-alpha band, and IAF to (IAF + 1) as upper-alpha band^60^. For each child, mean theta power in the frontal midline region (pooled from AFz plus 4 surrounding electrodes) was calculated for each of the four conditions separately and log-transformed to approach a normal distribution across children^23,61^. Mean power in the lower- and upper-alpha bands in the posterior region^30,31^ (pooled from Pz, P3, P4 and 15 surrounding electrodes) was calculated and log-transformed as well for each condition and child.

## Supplementary Information

Supplementary Information.

## Data Availability

The data that support the findings of this study are available from the corresponding author upon reasonable request.

## References

[1] Norton ES, Beach SD, Gabrieli JDE (2015). Neurobiology of dyslexia. Curr. Opin. Neurobiol..

[2] Langer N, Benjamin C, Minas J, Gaab N (2015). The neural correlates of reading fluency deficits in children. Cereb. Cortex.

[3] Kovelman I (2012). Brain basis of phonological awareness for spoken language in children and its disruption in dyslexia. Cereb. Cortex.

[4] Follmer DJ (2018). Executive function and reading comprehension: A meta-analytic review. Educ. Psychol..

[5] Baddeley A (1992). Working memory. Science.

[6] Jacob R, Parkinson J (2015). The potential for school-based interventions that target executive function to improve academic achievement: A review. Rev. Educ. Res..

[7] Melby-Lervåg M, Lyster S-AH, Hulme C (2012). Phonological skills and their role in learning to read: A meta-analytic review. Psychol. Bull..

[8] Chung KKH, McBride-Chang C (2011). Executive functioning skills uniquely predict Chinese reading. J. Educ. Psychol..

[9] Welsh JA, Nix RL, Blair C, Bierman KL, Nelson KE (2010). The development of cognitive skills and gains in academic school readiness for children from low-income families. J. Educ. Psychol..

[10] Chiappe P, Siegel LS, Hasher L (2000). Working memory, inhibitory control, and reading disability. Mem. Cogn..

[11] Peng P, Wang C, Tao S, Sun C (2017). The deficit profiles of Chinese children with reading difficulties: A meta-analysis. Educ. Psychol. Rev..

[12] Reiter A, Tucha O, Lange KW (2005). Executive functions in children with dyslexia. Dyslexia.

[13] Gibson EJ, Pick A, Osser H, Hammond M (1962). The role of grapheme-phoneme correspondence in the perception of words. Am. J. Psychol..

[14] McBride-Chang C, Chung KKH, Tong X (2011). Copying skills in relation to word reading and writing in Chinese children with and without dyslexia. J. Exp. Child Psychol..

[15] Huang HS, Hanley JR (1995). Phonological awareness and visual skills in learning to read Chinese and English. Cognition.

[16] Liu D, Chen X, Chung KKH (2015). Performance in a visual search task uniquely predicts reading abilities in third-grade Hong Kong Chinese children. Sci. Stud. Read..

[17] Haatveit BC (2010). The validity of d prime as a working memory index: Results from the ‘Bergen n-back task’. J. Clin. Exp. Neuropsychol..

[18] Pavlov YG, Kotchoubey B (2020). Oscillatory brain activity and maintenance of verbal and visual working memory: A systematic review. Psychophysiology.

[19] Onton J, Delorme A, Makeig S (2005). Frontal midline EEG dynamics during working memory. Neuroimage.

[20] Michels L, Moazami-Goudarzi M, Jeanmonod D, Sarnthein J (2008). EEG alpha distinguishes between cuneal and precuneal activation in working memory. Neuroimage.

[21] Gevins A, Smith ME, McEvoy L, Yu D (1997). High-resolution EEG mapping of cortical activation related to working memory: Effects of task difficulty, type of processing, and practice. Cereb. Cortex.

[22] Haier RJ (1988). Cortical glucose metabolic rate correlates of abstract reasoning and attention studied with positron emission tomography. Intelligence.

[23] Maurer U (2015). Frontal midline theta reflects individual task performance in a working memory task. Brain Topogr..

[24] Sternberg S (1966). High-speed scanning in human memory. Science.

[25] Brzezicka A (2019). Working memory load-related theta power decreases in dorsolateral prefrontal cortex predict individual differences in performance. J. Cogn. Neurosci..

[26] Klimesch W, Sauseng P, Hanslmayr S (2007). EEG alpha oscillations: The inhibition–timing hypothesis. Brain Res. Rev..

[27] Grabner RH, Fink A, Stipacek A, Neuper C, Neubauer AC (2004). Intelligence and working memory systems: Evidence of neural efficiency in alpha band ERD. Cogn. Brain Res..

[28] Güntekin B (2020). Theta and alpha oscillatory responses differentiate between six-to seven-year-old children and adults during successful visual and auditory memory encoding. Brain Res..

[29] Martínez-Briones BJ, Fernández-Harmony T, Garófalo Gómez N, Biscay-Lirio RJ, Bosch-Bayard J (2020). Working memory in children with learning disorders: An EEG power spectrum analysis. Brain Sci..

[30] Hu Z (2019). Working memory capacity is negatively associated with memory load modulation of alpha oscillations in retention of verbal working memory. J. Cogn. Neurosci..

[31] Sander MC, Werkle-Bergner M, Lindenberger U (2012). Amplitude modulations and inter-trial phase stability of alpha-oscillations differentially reflect working memory constraints across the lifespan. Neuroimage.

[32] Kraft I (2016). Predicting early signs of dyslexia at a preliterate age by combining behavioral assessment with structural MRI. Neuroimage.

[33] Puolakanaho A (2007). Very early phonological and language skills: Estimating individual risk of reading disability. J. Child Psychol. Psychiatry.

[34] Jensen O, Gelfand J, Kounios J, Lisman JE (2002). Oscillations in the alpha band (9–12 Hz) increase with memory load during retention in a short-term memory task. Cereb. Cortex.

[35] Beneventi H, Tønnessen FE, Ersland L, Hugdahl K (2010). Executive working memory processes in dyslexia: Behavioral and fMRI evidence. Scand. J. Psychol..

[36] Vasic N, Lohr C, Steinbrink C, Martin C, Wolf RC (2008). Neural correlates of working memory performance in adolescents and young adults with dyslexia. Neuropsychologia.

[37] Neubauer AC, Fink A (2009). Intelligence and neural efficiency. Neurosci. Biobehav. Rev..

[38] Larson GE, Haier RJ, LaCasse L, Hazen K (1995). Evaluation of a “mental effort” hypothesis for correlations between cortical metabolism and intelligence. Intelligence.

[39] Jaušovec N, Jaušovec K (2004). Differences in induced brain activity during the performance of learning and working-memory tasks related to intelligence. Brain Cogn..

[40] Gärtner M, Rohde-Liebenau L, Grimm S, Bajbouj M (2014). Working memory-related frontal theta activity is decreased under acute stress. Psychoneuroendocrinology.

[41] Zhang D, Zhao H, Bai W, Tian X (2016). Functional connectivity among multi-channel EEGs when working memory load reaches the capacity. Brain Res..

[42] Pan J, Cui X, McBride C, Shu H (2020). An investigation of the bidirectional relations of word reading to timed visual tasks involving different levels of phonological processing in Chinese. Sci. Stud. Read..

[43] Smith-Spark JH, Fisk JE (2007). Working memory functioning in developmental dyslexia. Memory.

[44] Swanson HL, Sachse-Lee C (2001). A subgroup analysis of working memory in children with reading disabilities: Domain-general or domain-specific deficiency?. J. Learn. Disabil..

[45] McBride-Chang C (2011). Visual spatial skill: A consequence of learning to read?. J. Exp. Child Psychol..

[46] Liu C, Chung KKH, Fung WK (2019). Bidirectional relationships between children’s executive functioning, visual skills, and word reading ability during the transition from kindergarten to primary school. Contemp. Educ. Psychol..

[47] Ho CS-H, Chan DW, Chung KKH, Lee S-H, Tsang S-M (2007). In search of subtypes of Chinese developmental dyslexia. J. Exp. Child Psychol..

[48] Wang L-C, Yang H-M (2014). Classifying Chinese children with dyslexia by dual-route and triangle models of Chinese reading. Res. Dev. Disabil..

[49] Gray S (2019). Working memory profiles of children with dyslexia, developmental language disorder, or both. J. Speech Lang. Hear. Res..

[50] Cowan N (2017). Short-term memory in childhood dyslexia: Deficient serial order in multiple modalities. Dyslexia.

[51] Rispens J, Baker A (2012). Nonword repetition: The relative contributions of phonological short-term memory and phonological representations in children with language and reading impairment. J. Speech Lang. Hear. Res..

[52] Carroll JM, Solity J, Shapiro LR (2016). Predicting dyslexia using prereading skills: The role of sensorimotor and cognitive abilities. J. Child Psychol. Psychiatry.

[53] Ho CS-H, Chan DW-O, Chung KKH, Tsang S-M, Lee S-H, Fong CY-C (2016). The Hong Kong Test of Specific Learning Difficulties in Reading and Writing for Primary School Students.

[54] Chung KKH (2017). Understanding developmental dyslexia in Chinese: Linking research to practice. Asia Pac. J. Dev. Differ..

[55] Raven J (2000). The Raven’s Progressive Matrices: Change and stability over culture and time. Cogn. Psychol..

[56] Leung, M. T. & Lee, A. *The Hong Kong corpus of primary school Chinese*. Paper presented at the ninth meeting of the International Clinical Phonetics and Linguistics Association, Hong Kong, China (2002).

[57] Jung T-P (2000). Removal of eye activity artifacts from visual event-related potentials in normal and clinical subjects. Clin. Neurophysiol..

[58] Perrin F, Pernier J, Bertnard O, Giard MH, Echallier JF (1987). Mapping of scalp potentials by surface spline interpolation. Electroencephalogr. Clin. Neurophysiol..

[59] Lehmann D, Skrandies W (1980). Reference-free identification of components of checkerboard-evoked multichannel potential fields. Electroencephalogr. Clin. Neurophysiol..

[60] Doppelmayr M, Klimesch W, Pachinger T, Ripper B (1998). Individual differences in brain dynamics: Important implications for the calculation of event-related band power. Biol. Cybern. Heidelb..

[61] Gasser T, Bächer P, Möcks J (1982). Transformations towards the normal distribution of broad band spectral parameters of the EEG. Electroencephalogr. Clin. Neurophysiol..

