# Supplementary material for: Behavioral and neurophysiological aspects of working memory impairment in children with dyslexia
Source: Sci Rep. 2022 Jul 22;12:12571. doi: 10.1038/s41598-022-16729-8 (PMC9307804; doi:10.1038/s41598-022-16729-8)
Supplement: Supplementary file 1 — Supplementary Information. [file 41598_2022_16729_MOESM1_ESM.pdf]

Supplemental Material for *Scientific Reports*

Table S1 lists the thirty 3 × 3 checkerboard patterns used in the visual 1-back and 2-back tasks. We did not use 60 different patterns, as it would yield too many similar patterns (e.g., 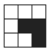 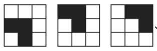) making the task too difficult. In both 1-back and 2-back tasks, the patterns were sequenced in a way that each pair of consecutive patterns did not look similar (e.g., 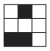 would not appear immediately before or after 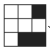), except for the targets in the 1-back task.

Table S1: *Thirty Checkerboard Patterns Used in the Visual n-Back Tasks*

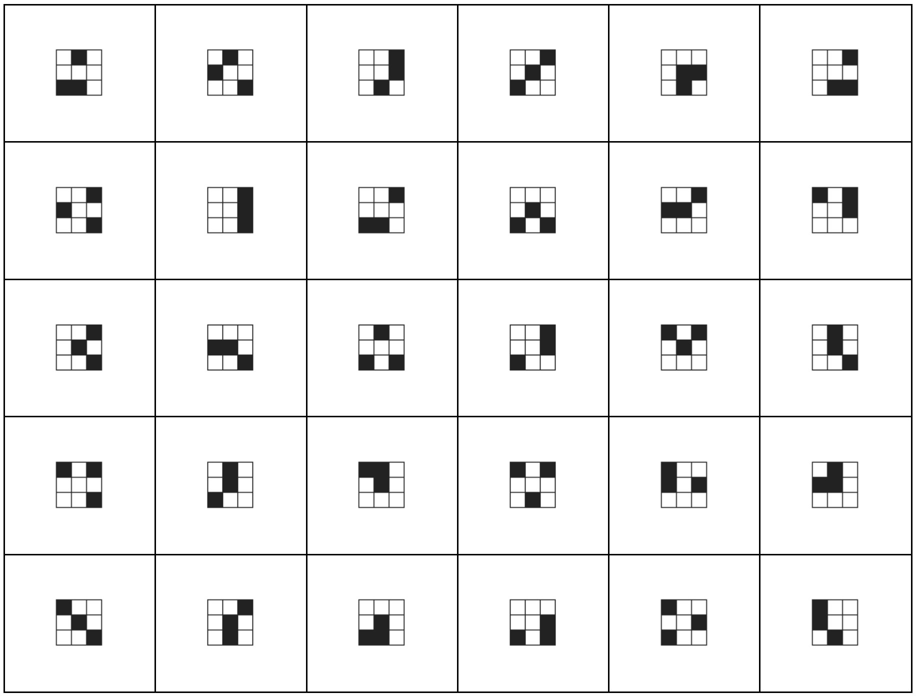

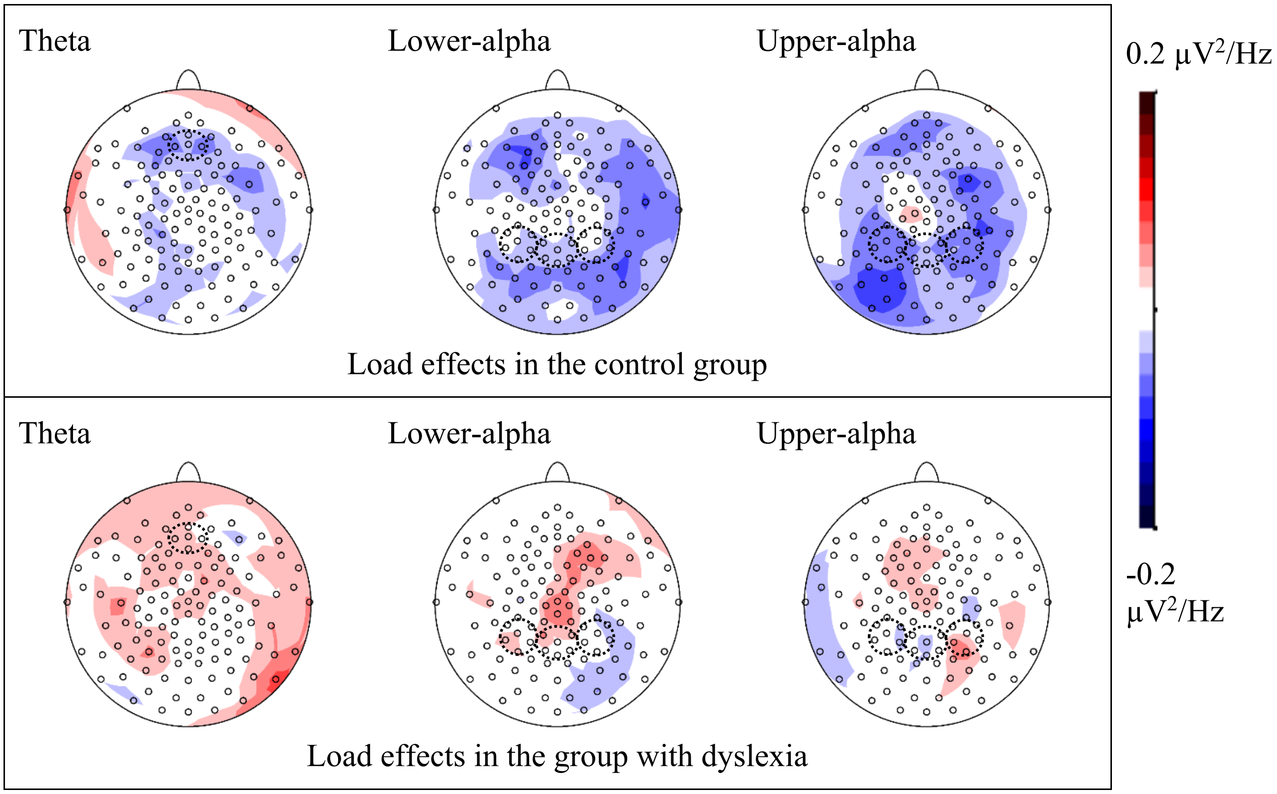

Figure S1. The Load effects (2-back – 1-back) on the log-transformed power of theta, lower-alpha, and upper-alpha bands during working memory maintenance. Data were collapsed across the two types of working memory tasks (verbal and visual). Frontal midline theta power was pooled from AFz plus 4 surrounding electrodes; posterior alpha power was pooled from Pz, P3, P4 and 15 surrounding electrodes (selected electrodes are enclosed by the dashed circles).

(a) Model 1: non-verbal intelligence

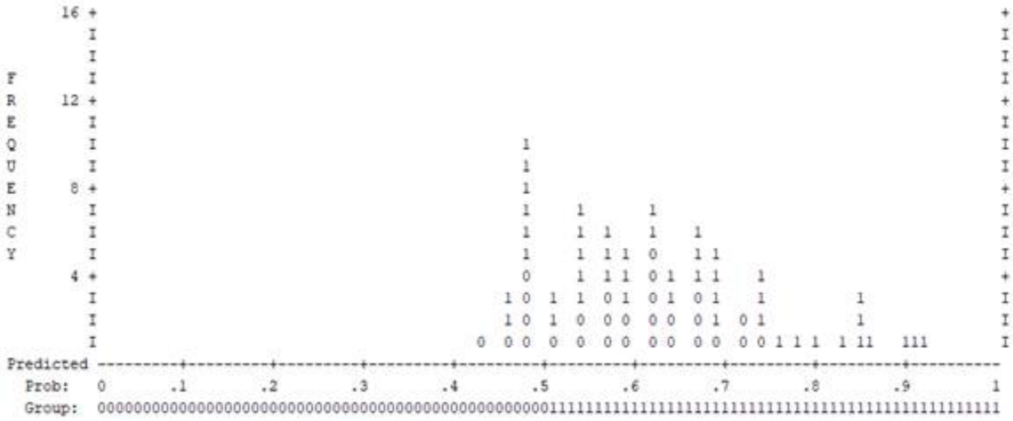

(b) Model 2: non-verbal intelligence, RT(visual 1-back), *d'*(verbal 2-back)

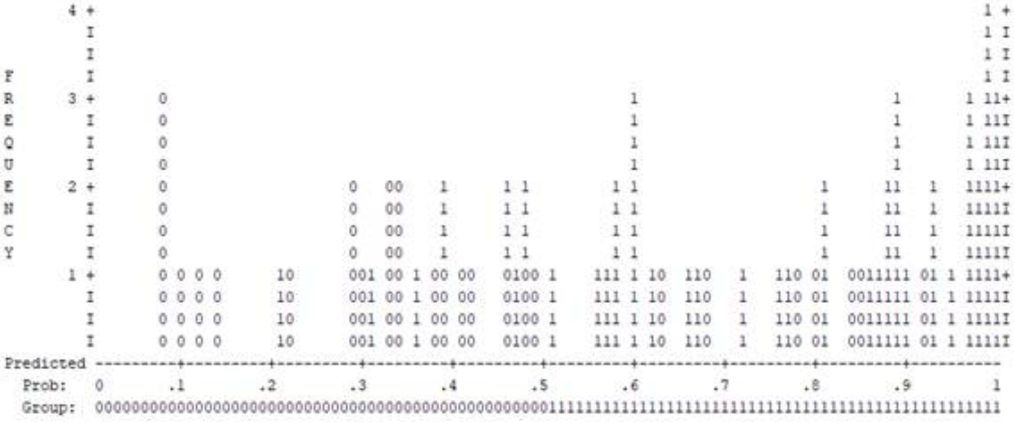

(c) Model 3: non-verbal intelligence, RT(visual 1-back), *d'*(verbal 2-back), theta(verbal 2-back), upper-alpha(visual 1-back)

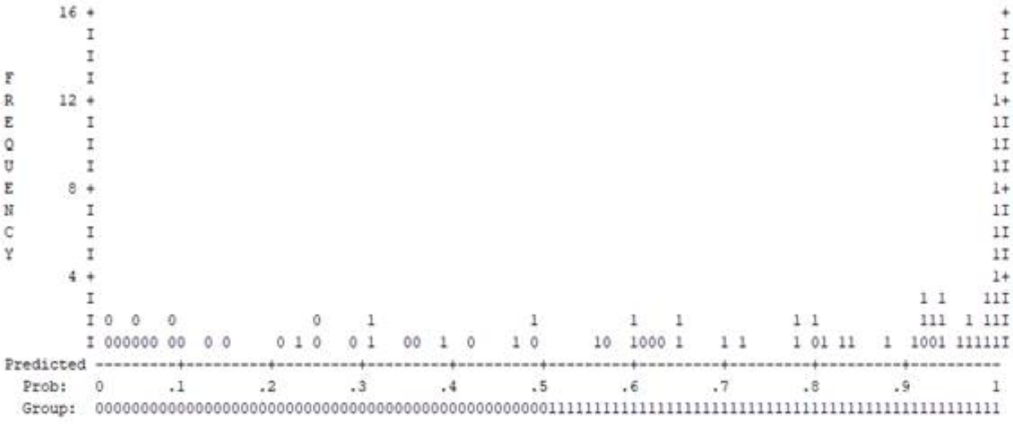

Figure S2. Classification plots of each model in the first logistic regression analysis. Group 0 = control; Group 1: dyslexic. The symbols inside the plots indicate the observed membership of each participant, while the x-axis value denotes the predicted probability of having dyslexia (0.50 as the cut value).

Additional logistic regression analysis after matching non-verbal intelligence

To further examine the predictive role of WM measures in dyslexia without any confounding effect of non-verbal intelligence, we conducted additional logistic regression analysis on the control group and a subsample of the group with dyslexia (i.e., excluding those who scored 21 or lower in non-verbal intelligence), whose non-verbal intelligence scores were comparable ($p = 0.860$). In this subsample, the two groups did not differ significantly in gender, age, grade, maternal or paternal education level, or monthly family income (ps ≥ 0.171). The following variables from the WM tasks were entered by block into logistic regression models as predictors of dyslexia: 1) Block 1 included general control variables: age, grade, non-verbal intelligence; 2) Block 2 included 8 behavioral variables: reaction time and *d* prime in each of the four conditions; and 3) Block 3 included 12 neurophysiological variables: log-transformed frontal midline theta, posterior lower- and upper-alpha power in each of the four conditions. The forward Wald method was adopted in each block.

Table S2 shows the logistic regression models generated in each block. In Block 1, no variable was a significant predictor. In Block 2, two behavioral variables (i.e., verbal 2-back RT, visual 1-back d') were entered (ps ≤ 0.013). Model 2b significantly improved prediction of dyslexia relative to a null model ($\chi^2_{(2)} = 19.37$, $p < 0.001$). The classification accuracy increased from 55.6% to 74.6% (dyslexic: 82.9%; control: 64.3%). In Block 3, two neurophysiological variables (i.e., log-transformed frontal midline theta and posterior upper-alpha in the verbal 2-back condition) were further entered (ps ≤ 0.043), while the two behavioral variables remained significant (ps ≤ 0.018). Model 3b significantly improved prediction of dyslexia relative to Model 2b ($\chi^2_{(2)} = 10.60$, $p = 0.005$). The classification accuracy further increased from 74.6% to 77.8% (dyslexic: 82.9%; control: 71.4%), and Nagelkerke's R^2 improved from 0.354 to 0.507.

To sum up, after matching the non-verbal intelligence of the two groups, behavioral and neurophysiological measures of WM still significantly and uniquely predicted dyslexia. Figure S3 displays the scatterplots of the two groups in the subsample, showing each significant predictor of dyslexia (y axis) as a function of non-verbal intelligence (x axis).

Table S2: *Parameter Estimates, Standard Errors, and Statistical Significance in the Logistic Regression Analyses of Factors Associated with Dyslexia after* ***the Non-verbal Intelligence of the Two Groups Being Matched***

	B	*SE*	Wald	*p*	χ^2	*df*	Nagelkerke's R^2	Classification Accuracy
Model 1b					--	--	--	55.6%
No variable included	--	--	--	--				
Model 2b					19.37***	2	0.354	74.6%
RT(verbal 2-back)	0.003	0.001	6.114	0.013*				
d'(visual 1-back)	-0.791	0.294	7.231	0.007**				
Model 3b					29.97***	4	0.507	77.8%
RT(verbal 2-back)	0.004	0.002	5.617	0.018*	(Model 3b vs: Model 2b: $\chi^2_{(2)} = 10.60$, $p = 0.005$)			
d'(visual 1-back)	-0.920	0.334	7.575	0.006**				
Theta(verbal 2-back)	4.388	2.168	4.096	0.043*				
Upper-alpha(verbal 2-back)	-3.780	1.432	6.970	0.008**				

*$p < 0.05$, **$p < 0.01$, ***$p < 0.001$.

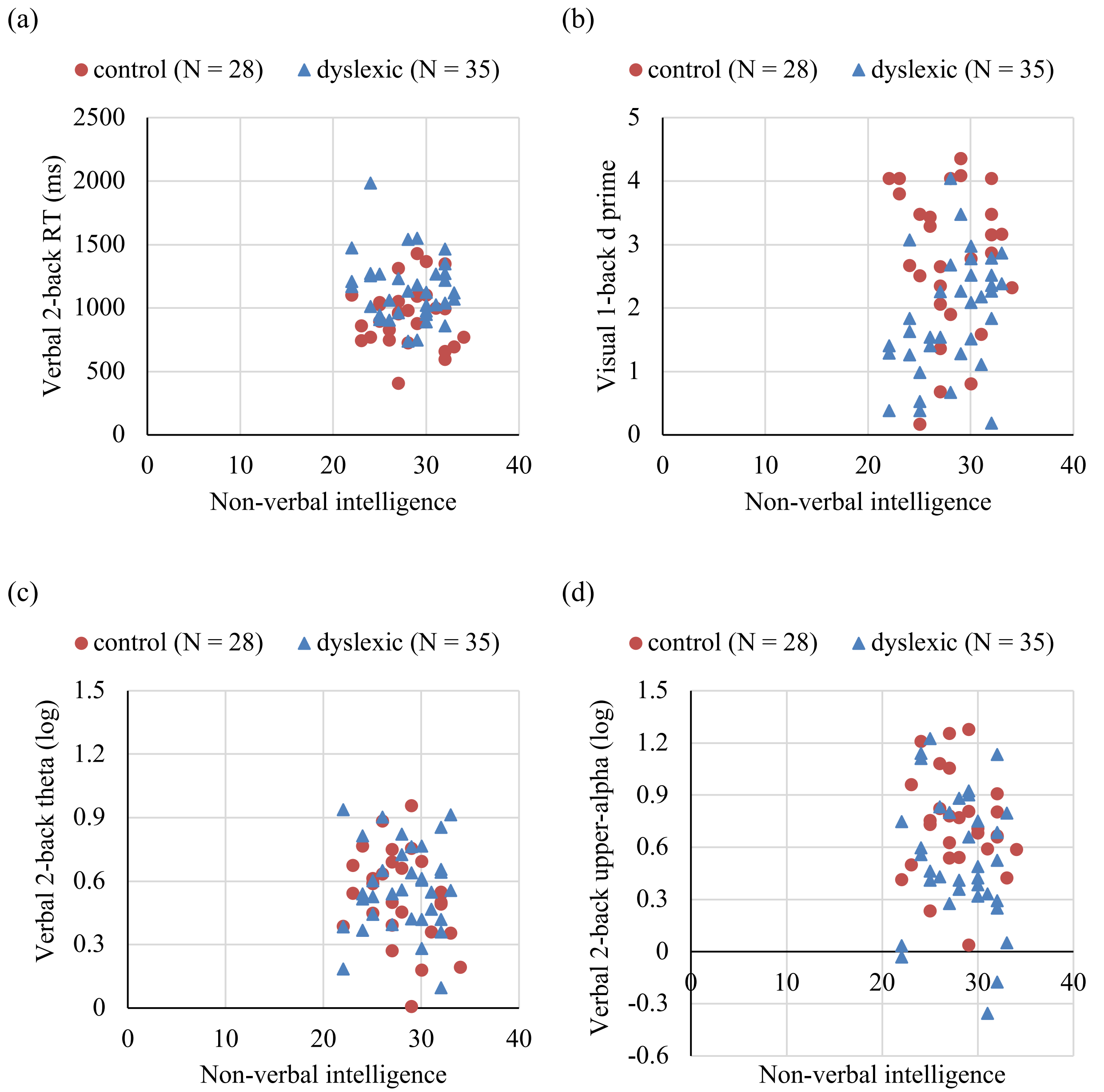

Figure S3. Scatterplots of the two groups showing each significant predictor of dyslexia (y axis) as a function of non-verbal intelligence (x axis), after matching the non-verbal intelligence of the two groups. Significant predictors included (a) reaction time in the verbal 2-back condition, (b) *d′* in the visual 1-back condition, (c) log-transformed frontal midline theta in the verbal 2-back condition, and (d) log-transformed posterior upper-alpha in the verbal 2-back condition.
